# Shortening Turnaround Times for Newborn HIV Testing in Rural Tanzania: A Report from the Field

**DOI:** 10.1371/journal.pmed.1001897

**Published:** 2015-11-03

**Authors:** Sabina Manumbu, Luke R. Smart, Anna Mwale, Kedar S. Mate, Jennifer A. Downs

**Affiliations:** 1 Bugando Medical Centre Care and Treatment Center/Baylor College of Medicine Children’s Foundation Tanzania, Mwanza, Tanzania; 2 Department of Internal Medicine, Catholic University of Health & Allied Sciences–Bugando, Mwanza, Tanzania; 3 Center for Global Health, Weill Cornell Medical College, New York, New York, United States of America; 4 Department of Internal Medicine, Weill Cornell Medical College, New York, New York, United States of America; 5 Institute for Healthcare Improvement, Cambridge, Massachusetts, United States of America

## Abstract

Jennifer Downs and colleagues describe a low-tech performance improvement project to improve newborn HIV testing turnaround times.

**Summary Points**

- Early diagnosis of infants infected perinatally with HIV in sub-Saharan Africa depends on completion of multiple steps for results to reach an infant’s caregivers in a timely manner.
- This project, designed by a pediatric HIV nurse in Tanzania, applied sequential quality improvement interventions to reduce the lengthy turnaround time between dried blood collection from exposed infants and return of test results.
- Low-cost interventions successfully reduced the turnaround time from 55 to 38 days, and increased the number of results given to caregivers to >90%.
- This work can serve as a model for implementation to improve identification of HIV-infected infants in other resource-poor settings.

## Introduction

Newborns infected with HIV before, during, or shortly after delivery have high mortality, with 50% dying before one year of age, and 20% of these early deaths occurring between the first and third months of life [1]. Early diagnosis and treatment reduces mortality by up to 76% [2], but neonatal diagnosis is difficult. Antibody-based rapid HIV tests frequently yield false positive results because of transplacental transfer of maternal antibodies that can persist in the child’s circulation for up to 18 months [3]. For this reason, polymerase chain reaction (PCR)-based viral nucleic acid tests are recommended instead [4]. At present, viral DNA or RNA can only be identified in a laboratory with the technical capacity to perform PCR.

The demonstration that HIV RNA and DNA can be detected in dried blood spots (DBS) revolutionized newborn HIV testing in resource-poor areas in which laboratory facilities are limited [5]. Unlike serum samples that must either be tested within hours of collection or frozen for transport, DBS can be stored in warm, humid climates and later transported to reference laboratories for testing while still yielding accurate results [6]. These findings led the World Health Organization to endorse DBS testing as the single screening tool for all infants born to mothers with HIV infection or unknown HIV status [4]. Given that DBS are additionally now being used for viral load monitoring, drug resistance genotyping, and even neonatal screening for hereditary diseases [7–9], ensuring the feasibility and reliability of this system is crucial.

In northwestern Tanzania, where we work, Bugando Medical Center (BMC) serves as the reference laboratory for early infant diagnosis (EID) of HIV-exposed children. The laboratory’s EID program was first piloted in 2006 [10]. BMC’s laboratory serves a population of 13 million people, receiving DBS from 96 clinics in the seven regions surrounding the medical center.

The lead investigator for this project (SM) is a pediatric nurse providing primary care to HIV-infected children at a clinic in Magu, Tanzania, located approximately 70 kilometers east of the Bugando laboratory. She grew frustrated with the protracted turnaround time (TAT) for results of DBS collected from HIV-exposed infants attending the clinic. DBS collected during infants’ six-week immunization visits were still pending at children’s subsequent ten-week visits. Sometimes mothers never received their infants’ results. These delays in diagnosis led to preventable deaths, particularly given the approximately 30% mortality of perinatally-infected infants during the first six months of life [1] and the advanced stage of HIV disease that affects approximately half of HIV-infected infants who do not start antiretroviral therapy (ART) before 12 weeks of age [11]. She therefore designed a systems improvement project to identify and address the root causes of these delays.

## Systems Improvement Project Methodology

In the project’s first phase, each step in the EID DBS process was carefully mapped. Five steps in the process between collection of a DBS and reporting of the result to a caregiver were identified. Each time a DBS was collected, infant demographics, date of collection, and date of shipment to Bugando were recorded consecutively in a log book in Magu. When the DBS result was returned, nurses in Magu used the lab report to fill in the dates for the remaining steps in the process.

The study’s lead investigator collected this data from the handwritten log book and entered it into Microsoft Excel. Minor errors in dates with an obvious alternative were corrected (e.g., correct month and day but wrong year, or aberrant month in an otherwise consecutive series of dates). Ambiguous dates without an obvious alternative were recorded as missing data. The duration of each step was calculated based on the available (non-missing) data.

**Fig 1:**
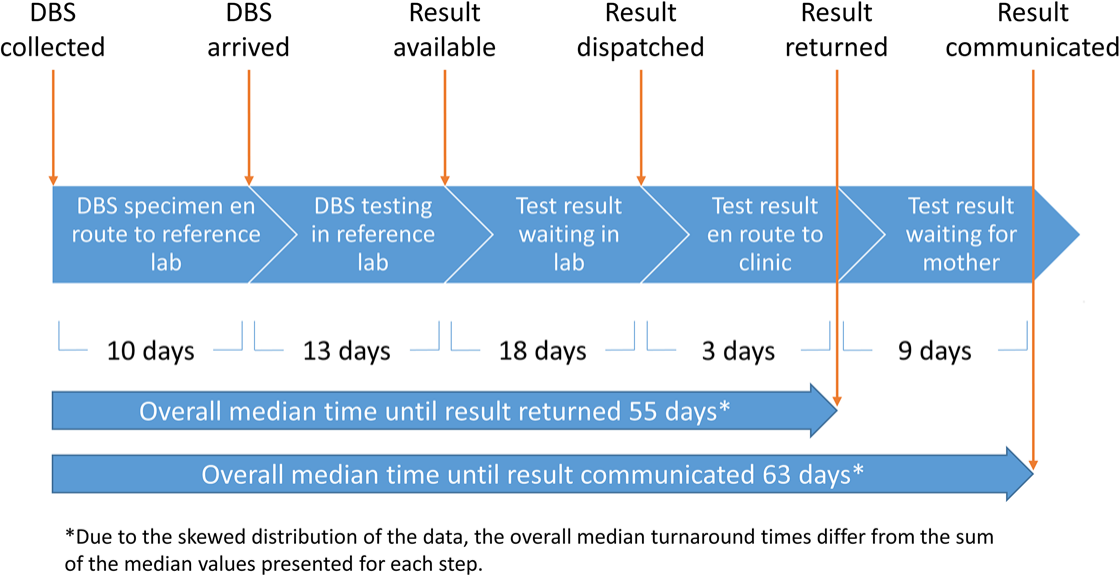
Flow diagram of steps required for rural dried blood spot HIV viral load testing and median times to completion at baseline.

Two significant clinical process endpoints were identified: (1) TAT between DBS collection and return of test result to the Magu clinic (when the result is available to the clinician and the local staff) and (2) TAT between DBS collection and communication of test result to the infant’s caregiver. **Fig 1** demonstrates the five steps in this process and lists the median number of days between each step during a baseline assessment in 2011.

In the second phase, the team implemented sequential interventions to decrease the time of individual steps. After each intervention, the team measured the resultant duration of each step as well as the cumulative TAT between DBS collection and the two designated endpoints. Consecutive interventions were designed and implemented based on observations and experience from prior interventions using a systems improvement method based on the Plan-Do-Study-Act model [12].

Data analysis was performed using Stata Version 13 (College Station, Texas). Due to non-normal distribution of the data, medians and interquartile ranges (IQRs) were calculated. Overall differences between groups were determined using the Kruskal-Wallis test. If the *p*-value was less than 0.10, we subsequently performed between-group comparisons using the Wilcoxon rank-sum test.

## Results

Between July 2011 and October 2013, 383 HIV-exposed infants were seen at Magu Health Centre and had DBS collected and sent to the Bugando reference laboratory for testing. Sixteen infants were entered into the Magu log book but had no further dates recorded and were excluded from further analysis. Infants were a median of 61.5 (IQR 39–133.5) days old at DBS collection. Baseline data were collected from July 2011 to January 2012 for 85 patients for whom DBS were sent, and these data are presented in **Fig 1**. The median overall baseline TAT from DBS collection to result availability at Magu was 55 (IQR 35–68.5) days, and TAT from DBS collection to result communication to mothers was 63 (IQR 35–88) days. Of note, due to the skewed distribution of the data, the total median turnaround times differ from the sum of the median values presented for each step in **Fig 1**.

We first attempted to decrease time between arrival of DBS at BMC and completion of testing (**Step 2**, **Fig 1**). The laboratory tests 44 samples per day in a single run on the COBAS Ampli-Prep/Taqman System (Basel, Switzerland) and receives 150–200 samples per day from health centers. Samples testing positive on the first run are repeated the following day. Due to staffing levels, the laboratory was unable to complete a second run per day on the machine. Our study team, therefore, turned its focus to other steps. Indeed, from 2011 to 2013 the median number of days that samples spent in the BMC laboratory actually increased slightly (**Table 1**).

**Table 1:** Median (interquartile range) number of days required for sequential steps for DBS testing over consecutive intervention periods.

	DBS specimen en route to reference lab	DBS testing in reference lab	Test result waiting in lab	Test result en route to clinic	Test result at clinic waiting for mother	TAT collection to Magu	TAT collection to mother
**Baseline [Jul 2011–Jan 2012]**	10 (4–17.5)	13 (11–17)	18 (10–34)	3 (3–7)	9 (3–31)	55 (35–68.5)	63 (35–88)
**Period 1 [Feb 2012–May 2012]: Phone voucher**	9 (5–18)	34 (24–40)	8 (5–13)	1 (0.5–1)	6.5 (1–8)	48 (43–65)	69 (60–82)
***p***-value for Wilcoxon rank sum test comparing Period 1 with Baseline	0.813	**<0.001**	**<0.001**	**<0.001**	**0.042**	0.867	0.199
**Period 2 [Jun 2012–Oct 2013]: Transport + home nurse**	6 (3–9)	21 (11–30)	14 (5–17)	0 (0–0.5)	6 (2–14)	38 (29–51)	58 (42–78)
***p***-value for Wilcoxon rank sum test comparing Period 2 with Baseline	**0.001**	**0.001**	**<0.001**	**<0.001**	0.072	**<0.001**	0.708
***p***-value for Wilcoxon rank sum test comparing Period 2 with Period 1	**<0.001**	**<0.001**	**0.014**	**<0.001**	0.561	**<0.001**	**0.012**
***p***-values for Kruskal-Wallis test comparing all three time periods	**<0.001**	**<0.001**	**<0.001**	**<0.001**	0.088	**<0.001**	0.057

Bold numbers indicate significant *p-*values <0.05.

We next attempted to decrease time between completion of testing at BMC and return of results to Magu (**Steps 3 and 4, Fig 1**). Beginning in February 2012, the study team provided a laboratory staff member with phone vouchers to call a nurse at Magu weekly and verbally relay results from the prior week. Verbal results were recorded in the Magu log book and acted upon, and they were subsequently confirmed when the written results arrived at Magu and were entered formally into the log book. This led to significant decreases in the time that the test result was ready in the lab waiting for transport (18 [IQR 10–34] days to 8 [IQR 5–13] days, *p* < 0.001) and to the amount of time taken for the test result to be transmitted from the lab to Magu (3 [IQR 3–7] days to 1 [IQR 0.5–1] day, *p* < 0.001). The overall TAT from DBS collection to return of result to Magu did not change significantly: a baseline value of 55 (IQR 38–68.5) days compared to 48 (IQR 43–65) days among patients who had blood spots collected from February to May 2012 (*p* = 0.87). The lack of significant decrease in cumulative TAT is likely attributable to the significant increase in time required for test performance in the reference laboratory (13 to 34 days, *p* < 0.001). The team additionally noted that 18/51 results (35.3%) that returned to Magu were not communicated to mothers, and room for improvement remained.

After June 2012, the team focused on two separate steps: time between collection of DBS and arrival at BMC (**Step 1, Fig 1)** and time between result arrival at Magu and result communication to mothers (**Step 5, Fig 1**). It was discovered that nurses at Magu were not sending DBS to Bugando until they had collected at least 3–5 samples. Instructions to send DBS as soon as possible after collection were reinforced, and nurses began to ask drivers of hospital vehicles between Magu and Bugando to carry blood spots to the laboratory whenever possible (usually 1–2 times weekly) rather than waiting to send samples in batches in a dedicated car from the Bugando HIV clinic. Median time between a DBS collection and arrival at Bugando decreased from 10 (IQR 4–17.5) days at baseline to 6 (IQR 3–9) days after June 2012 (*p* = 0.001). This led to an overall improvement in median TAT from 48 (IQR 43–65) days during the previous intervention period to 38 (IQR 29–51) days (*p* < 0.001).

After June 2012, the study team also partnered with an ongoing home-visit nurse program in Magu, in which clinic nurses spend one afternoon per week visiting homes in the community. The study team ensured that visiting nurses focused preferentially on visiting HIV-infected mothers to remind them to return to clinic to obtain their infants’ results. Additionally, nurses at the Magu clinic now leave a blank space on infants’ vaccination cards and fill in the DBS result when mothers obtain it. Taken together, these interventions led to consistent communication of results to mothers in 90.5% (95/105) of blood spot results that arrived at Magu after June 2012, compared with 47.2% (34/72) at baseline (*p* < 0.001).

**Fig 2:**
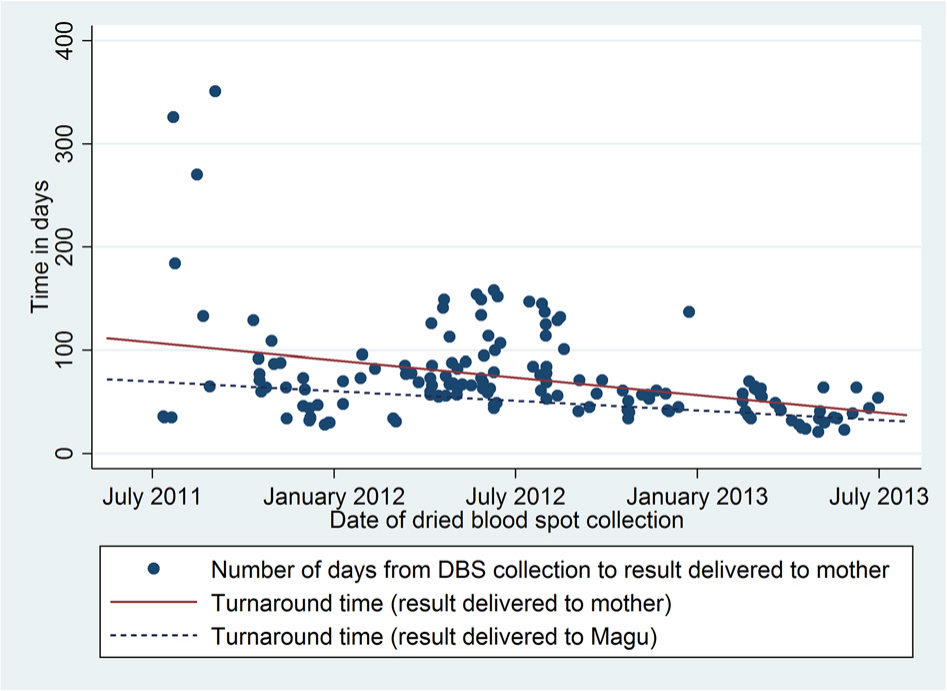
Turnaround times from dried blood spot collection until result available at health center (blue dashed line) or result given to mother (red solid line).

Since the project began, the overall TAT from DBS collection to return of results to Magu has decreased from a median of 55 (IQR 35–68.5) to 38 (IQR 29–51) days (*p* < 0.001, **Fig 2**). Dates of DBS collection and each sequential step continue to be recorded in the log book by the Magu nursing staff, and data from 2014 demonstrate that these levels have been sustained, with ongoing oversight from a nurse who visits Magu once monthly from Bugando but no further cost investment. The ongoing costs are weekly vouchers for phone calls (US$1 per week); other interventions have built on and/or redirected existing systems at negligible incremental cost. For example, the visiting nurse program is not a new program, but our intervention reprioritized the goals of the existing program. Since the start of this project, 18 HIV-infected infants’ mothers have been notified of their results, and these babies have been started on lifesaving antiretroviral therapy.

## Project Assessment and Discussion

Our work has demonstrated that significant improvements can be made in the implementation of neonatal HIV testing in a rural setting with dedicated attention and application of a simple, locally-driven, systems improvement methodology. During this two-year project, the study team’s interventions decreased DBS TAT from 55 to 38 days and improved result communication rates to mothers from 47% to 91%. These interventions required minimal incremental cost in the study setting and have been sustained. Our study team has now presented our experience to other nurses providing primary pediatric care in rural clinics in our region to equip them to devise their own locally-relevant solutions to address implementation problems at their respective health centers.

A major strength of our work is that it models the critical role of quality-improvement interventions within HIV programming, and particularly how such interventions can and often should be driven by local health center staff. Health workers caring for patients in rural settings have first-hand knowledge of system breakdowns and, often, can propose innovative, low-cost, sustainable ideas for improvement if given the opportunity to do so. In our case, our principal investigator initially sought to change procedures in the reference laboratory (largely beyond her control). When that proved impossible, she subsequently devised a series of simple solutions that, taken together, shortened the TAT by approximately 17 days, bringing major health benefits to her patients and empowerment to local staff. Such quality-improvement work, in which locally-devised innovations promote “buy-in” and rejuvenation for overburdened staff, was recognized as a key factor in multiple successful quality-improvement projects in South Africa [13].

To the best of our knowledge, no studies have been published on small-scale, low-cost systems improvement projects for EID such as ours. Three countries have published marked improvements in their EID TAT after large-scale technological or logistical investment. Rwanda made multiple changes to their system, including integrating EID into the immunization program, instituting a national pickup system to collect samples twice weekly from facilities, and implementing an automatic SMS system that sends results back to the mobile phone of the provider who ordered the test and the laboratory technician at the facility where the DBS originated. This package of interventions decreased median TAT from DBS collection to return of results to the clinic from 144 to 20 days [14]. Uganda invested in a national pickup system, providing a motorbike and driver for each of their collection hubs and a national SMS printer system that sends results back to the hubs automatically from the reference laboratory. Uganda’s TAT from DBS collection to return of results decreased from 49 to 14 days [15]. Zambia piloted a similar automatic SMS system, and TAT from DBS collection to result communication to the mother dropped from 67 to 35 days [16].

In contrast to these large projects, our work, at a fraction of the cost, demonstrates the feasibility of local interventions in achieving TATs comparable to those reported through expensive, countrywide systems. It is our hope that other local clinics will use our work as a model to devise low-cost action plans relevant to their own contexts. In our case, simple changes such as writing reminders to obtain results on children’s health cards and providing a nurse with dedicated time to seek out caregivers who have not obtained their children’s results were effective interventions that required investment of relatively little money and time. Of note, Magu benefits from existing infrastructure, like the visiting nurse program and a close transportation connection with BMC, that might not be present in other settings. We outline key barriers and opportunities for change in **Table 2**. We recognize that this project’s focus on neonatal DBS testing represents only one aspect of highly complex prevention of mother-to-child transmission of HIV (PMTCT) programs. But improving this aspect of PMTCT programs needs to be a non-negotiable, urgent goal.

**Table 2:** Barriers to expeditious dried blood spot testing and potential solutions.

**Delayed transport from collection site to central laboratory**	
**Problems**	**Solutions**
1) Busy health center staff responsible for coordination of transport	1) Shift burden of collection of samples to be transported to a driver rather than health center staff
2) Health center staff trying to decrease costs by shipping more samples at once	2) Standardize and routinize specimen pickup at regular, frequent intervals
	3) Emphasize to health center staff the larger cost of seeking out patients later whose results are returned late
**Delayed transmission of results to health center**	
**Problems**	**Solutions**
1) No person in laboratory responsible for result transmission	1) Place log or register in laboratory requiring documentation of result transmission
2) Laboratory’s emphasis is maximizing number of test results rather than sending results	2) Implement measurement of time to result transmission in laboratory as a quality-control standard
	3) Subsidize phone/SMS communication or institute automated SMS transmission method
**Difficulty providing results to mothers/caregivers**	
**Problems**	**Solutions**
1) Caregivers do not understand urgency of obtaining results	1) Improve counseling provided to caregivers at time of dried blood spot collection
2) Many caregivers return routinely for vaccinations but not at other times	2) Ensure that home visit/outreach nurse reminds caregivers to return for results
3) Some caregivers grow frustrated with returning to clinic only to learn that results have still not arrived	3) Insert space for result on child’s health card so caregiver notices that it is incomplete

Despite these successes, our work also demonstrates the need for an ongoing discussion about the feasibility of relying on such a complex multi-step process for provision of laboratory testing in remote settings. Our local neonatal testing algorithm is a five-step process that works smoothly only when laboratory supply and functionality, transport, communication, and patient follow-up systems are simultaneously operational. A breakdown at any step creates an impasse and delays HIV diagnosis for hundreds of children in northwest Tanzania. In the face of these challenges, the growing optimization of point-of-care nucleic acid testing for infant HIV diagnosis offers hope that EID will eventually become a simple, streamlined service that provides same-day results, allowing earlier initiation of ART for infected infants and earlier cessation of ART for uninfected ones [17,18].

In conclusion, our work highlights the major importance, on both local and national scales, of working to improve the implementation of HIV programs with proven effectiveness. With the impending expansion of DBS testing for HIV viral load monitoring and genetic disease screening, our successes and challenges highlight the importance of a realistic assessment of this system’s limitations. Yet we hope that our experience also engenders hope that simple, practical interventions can have real impact on the health and well-being of patients in resource-poor settings. We continue to strive towards additional improvements in TAT, with particular focus on increasing the percentage and frequency of mothers returning for results by providing community education on the importance of EID. We are also now evaluating the percentage of infants who are ultimately started on lifesaving ART, which is the ultimate goal of efforts to expedite EID. We share our experience in hopes that we, other local clinicians, and national leaders will resolve to work together to streamline and/or de-centralize this system in order to make essential diagnostic testing more accessible.

## Supporting Information

S1 Data De-identified dataset recorded from Magu log book. (XLS)

## Abbreviations

ART: antiretroviral therapy
BMC: Bugando Medical Centre
DBS: dried blood spot
EID: early infant diagnosis
IQR: interquartile range
PCR: polymerase chain reaction
PMTCT: prevention of mother-to-child transmission
TAT: turnaround time
